# Correspondence

**Published:** 1901-04

**Authors:** I. J. Jones

**Affiliations:** Quarantine Department, Austin, Texas


### Correspondence.

QUARANTINE DEPARTMENT,
AUSTIN, TEXAS, April 8, 1901.

*Editor Texas Medical Journal.*

I take pleasure in answering your request for information of status of pending quarantine legislation.

The first measure introduced was the Strother bill in the House, providing that when counties fail, refuse or neglect to enforce quarantine against infectious diseases, that the Governor shall order the

State Health officer to administer the quarantine for such counties at the counties expense. This bill was substituted on motion of Mr. Heslup, by providing that the commissioners court shall be required to declare quarantine, as now provided by law, and the expense thereof should be paid two-thirds by the State and one-third by the county. The bill in this form passed and went to the Senate, where it has been amended back to its original shape, with the exception that counties bordering on the Rio Grande and Indian Territory shall be required to pay only one-half their quarantine expenses, the other half to be paid by the State. The bill is now before a free conference committee of the two houses.

Another bill was introduced into the House by Dr. Looney, which defines the offense of violation of quarantine and provides a penalty of from ten dollars to one thousand dollars for such violation. This bill also requires physicians to report all cases of infectious disease, or any disease resembling the same, to the county or State health officers within six hours after they shall have knowledge of the same, and provides the fine not to exceed one hundred dollars for violation of this provision. It also provides a penalty on common carriers for violation of the Governor's proclamation. This bill passed in the House without opposition and is pending in the Senate, and will be certainly passed.

Another bill has passed in the Senate and is pending in the House specifically authorizing the Governor to prescribe fees for the boarding and disinfecting of vessels entering the ports of Texas.

The compulsory vaccination bill was killed in the House Judiciary Committee No. 2, by vote of eight to seven. The friends of the bill were surprised at the strength it developed, and believe that if it could have been gotten before the House earlier in the session that it would have passed. Very truly,

I. J. JONES, M. D.

